# Treated Pierre Robin Sequence Using Placed Allogenic Acellular Bone Matrix and Mandibular Distraction Osteogenesis in the Neonate

**DOI:** 10.3389/fped.2022.890156

**Published:** 2022-05-23

**Authors:** Tao Han, Yi Ji, Jie Cui, Liangliang Kong, Lijun Shi, Jianbin Chen, Weimin Shen

**Affiliations:** Department of Burns and Plastic Surgery, Children's Hospital of Nanjing Medical University, Nanjing, China

**Keywords:** Pierre Robin, mandibular distraction osteogenesis, allogenic acellular bone matrix, neonates, complications

## Abstract

**Objective:**

The aim of the study was to report our experience with placed allogenic acellular bone matrix and mandibular distraction osteogenesis in Pierre Robin sequence (PRS), and explore the role of distraction in the osteogenesis of acellular bone.

**Materials and Methods:**

A total of 428 neonates with severe PRS managed with placing allogenic acellular bone and bilateral mandibular distraction osteogenesis were included in the study. The procedure included using oblique-shaped osteotomy, fixing bilateral mandibular distractor, instantly extending a 4–6 mm gap, and placing allogenic acellular bone into the gap. The length of allogenic acellular bone was 4–5 mm. Although the surgical techniques, distraction, and consolidation periods were similar, the allogenic acellular bone matrix we placed was quite different from the traditional distraction. With the technology we used, tracheal intubation could be immediately removed, thus quickly improving breathing conditions compared to traditional methods after the surgery. The jaw extending and oral feeding could begin on the 5th day. The jaw was extended 0.6 mm twice a day until the mandible was overcorrected by 20%.

**Results:**

All 428 cases included in this study were successfully extubated after the operation, and the difficulty in breathing was instantly relieved. Total mandibular distraction was 15–20 mm. Oral feeding was started at 6 h to 6 days postoperatively, while hospital stay ranged from 18 to 20 days postoperatively. No major complications were reported. Medium to long-term results was good. Mandibular distractors were removed after 3 months.

**Conclusions:**

Bilateral mandibular distraction osteogenesis combined with placing allogenic acellular bone in the neonate are safe and accurate procedures, which are the primary treatment options for cases of severe PRS. It can be considered that the tension of distraction can promote osteogenesis in acellular bone and thus improve distractive effect of the mandible.

## Introduction

Pierre Robin sequence (PRS, MIM number: 261800), a condition where baby is born with a small lower jaw, is typically described as micrognathia, glossopteris, and cleft palate (1). Neonates with PRS at birth present with micrognathia, difficulty feeding, and difficulty breathing. Micrognathia constricts the tongue, forcing it more backward and upward, thus resulting in obstruction of upper airway and forming a U-shaped cleft palate. If intervention with bilateral mandibular osteogeneses is not timely performed, tracheostomy needs to be performed to guarantee to breathe. Tracheostomy may be considered as a long-term solution, as children can breathe smoothly with a large pharyngeal cavity when they grow up. Distraction osteogenesis (DO) is an alternative treatment (2, 3) as in PRS, and mandible is distracted gradually under tension across a surgical osteotomy. Distracting the mandible does not immediately decrease dyspnea, as it takes 7–14 days for the difficulty in breathing to be relieved. Consequently, a new method is needed to solve the issue of instant extubating after the operation and improve airway condition.

In the present study, we described bilateral mandibular DO and placed allogenic acellular bone matrix between mandibular osteotomy in 428 patients with Pierre Robin sequence and severe airway obstruction, which immediately solved the airway problem.

## Patients and Methods

### Patients

A total of 428 neonates diagnosed with PRS and life-threatening with upper airway obstruction from January 2011 to January 2021, were included in the study. A multispecialty neonatal obstructive airway team at the Children's Hospital of Nanjing Medical University consisted of the physicians of SICU, neonatologist, pediatric anesthesiologist, pediatric otolaryngologist, and a pediatric plastic surgeon. 128 neonatal patients were seen primarily at the Children's Hospital of Nanjing Medical University, while 300 neonatal patients were referred from other institutions. Traditional symptomatic management failed in all cases, including nasopharyngeal airway intubation and prone positioning. All patients suffered with mandibular micrognathia, glossoptosis, and cleft palate. In all patients, their intermittent resting oxygen saturation levels were <80%. Anoxia, difficulty in feeding and other simultaneous diseases are shown in Table 1.

**Table 1:** Presentation of respiratory, feeding difficulties, and other deformities.

**Number**	***n***	**% Of all patients (*n* = 428)**
RD	428	100
Upper airway obstruction	417	97.4
Respiratory infection	410	95.7
Laryngomalacia	182	42.5
Tracheomalacia	76	17.7
Cardiovascular system	168	39.2
Central nervous system	108	25.2
Subglottic stenosis	28	6.5
Lower airway obstruction	22	5.1
Diaphragmatic hernia	12	2.8
Bilateral choanal atresia	5	1.1
Unilateral choanal atresia	1	0.2
FD	428	100
Intake problems	428	100
GERD	38	8.8
RD during feeding	428	100
Failure to thrive	189	44.2
Vomiting	116	27.1
Food allergy	14	3.3
Severe malnutrition	48	11.2

*RD, respiratory difficulties; FD, feeding difficulties; GERD, gastro-esophageal reflux disease*.

### Per-Operation Managements

Presence of breathing problems and other congenital anomalies were recorded in all patients. All patients underwent prone positioning, nasopharyngeal airway, and tracheal intubation (oral). Adequate nutrition was achieved by nasogastric gavage feeding in all patients. A craniofacial 3D CT scan and lateral X-ray film were performed before surgical planning to define the mandibular anatomy, and the distance between the post pharyngeal wall and lingual root was measured. The distance from post pharyngeal wall to lingual root <3 mm and dyspnea was considered as indications for operation (4). Of course, the decision to operate was also based on the patient's clinical status, such as feeding difficulty, the preoperative peripheral oxygen saturation <80%, and the failure of non-operative management.

### Operation Anesthesia

The operations were performed under general anesthesia. As ordinarily, tracheal intubation can be difficult to perform in these PRS patients, intubation with nasendoscopy was performed in all 428 patients.

### Operation

**Figure 1:**
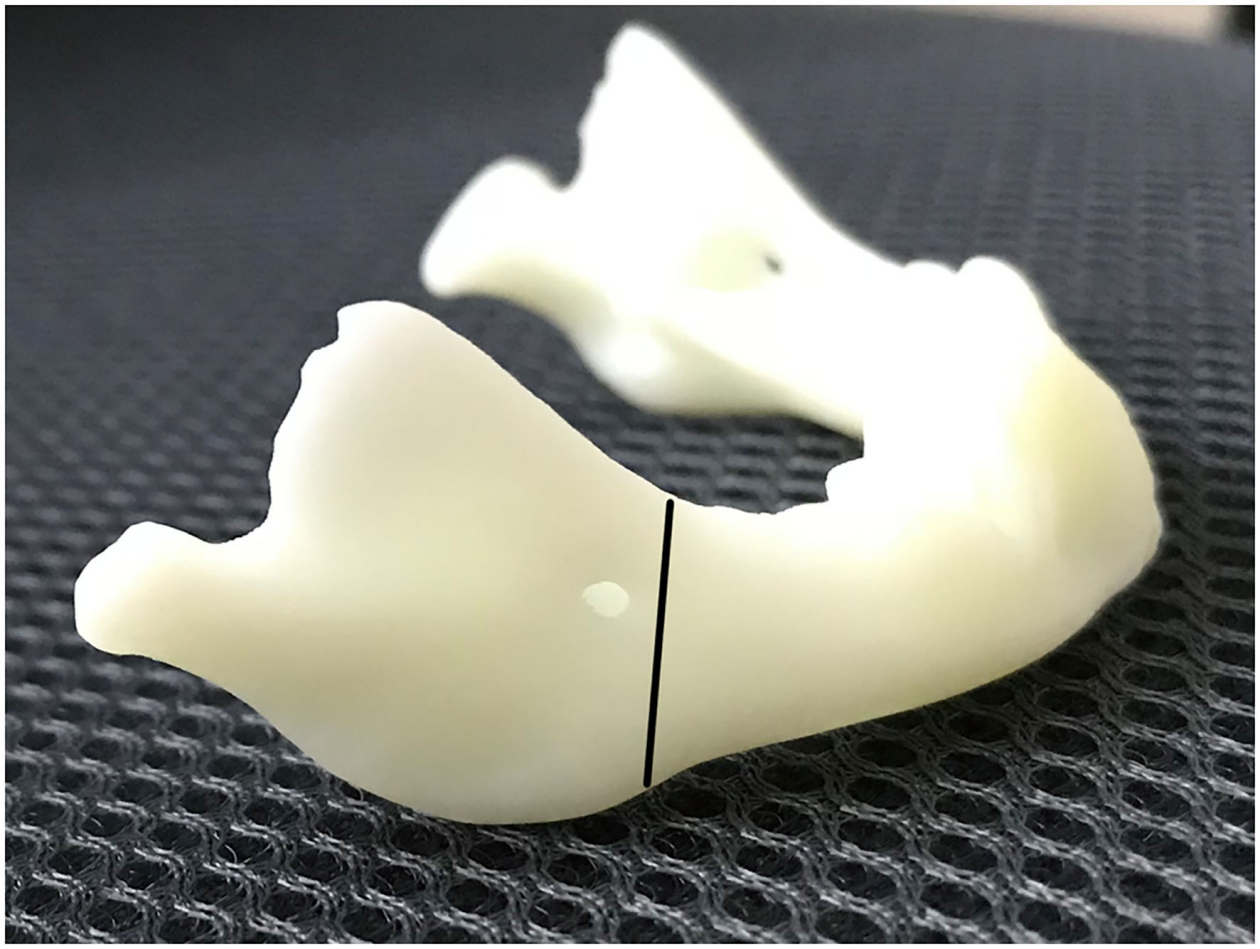
The oblique osteotomy is planned.

**Figure 2:**
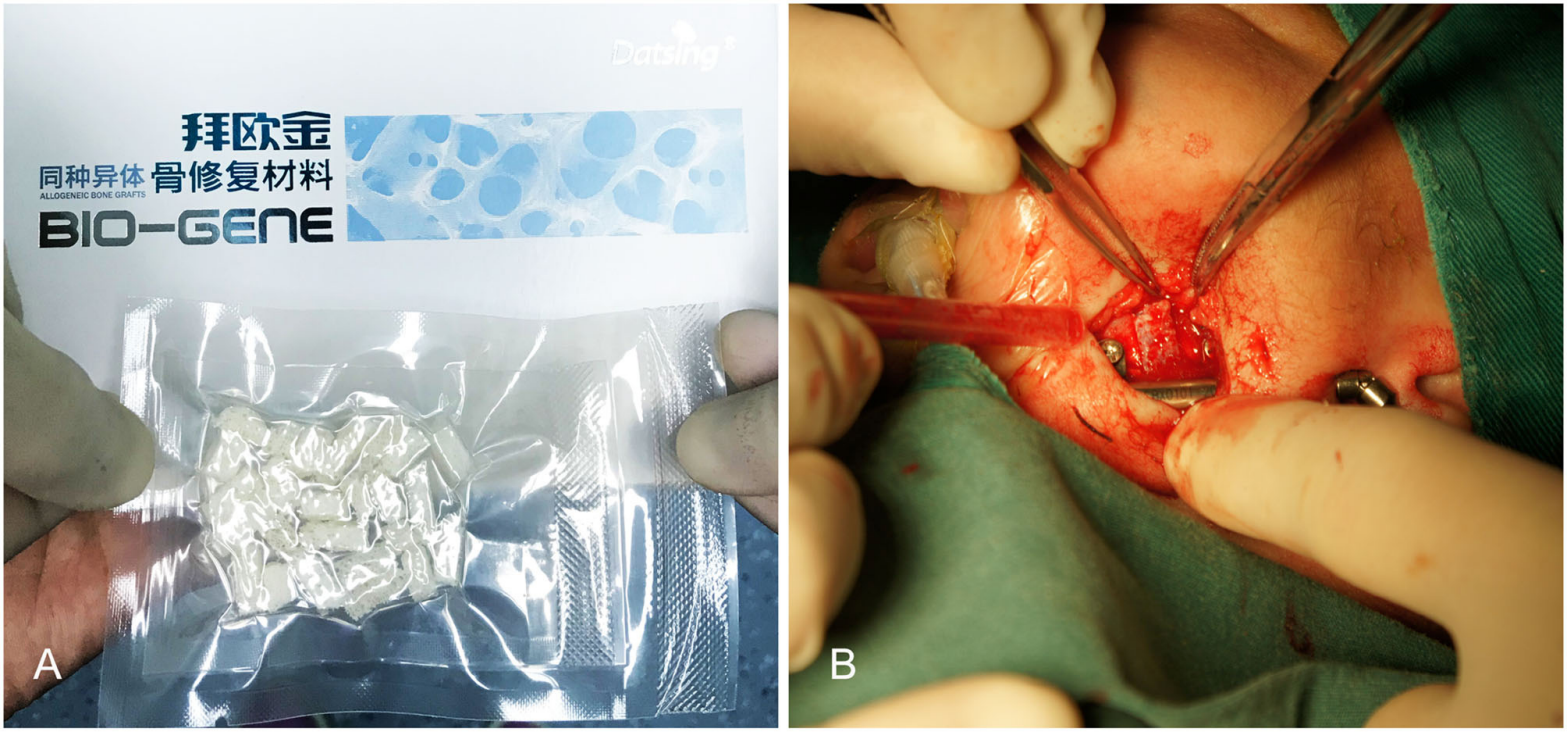
**(A)** Filling this gap with 0.5 mm allogenic acellular bone. **(B)** Making the two-side mandibular decellularized bone block clamping.

The skin incision was symmetrically placed approximately 15 or 20 mm to infra mandibular region. After the incision was made, the subcutaneous fat was cut and dissected from superficial to deep layer with a vessel clamp, dissecting to periosteal surface. Dissection was continued until the pterygomasseteric slang along the angle of the mandible, and inferior borders of the mandible were identified with a periosteal elevator. Gentle, blunt dissection was used until the pterygomasseteric sling muscle fibers remained, reducing the risk of injury to the marginal mandibular branch. Then we performed subperiosteal dissections along the buccal and cortices. The planned oblique osteotomy line was painted on the bone with a methylene blue marker pen, after which we performed the oblique osteotomy (Figure 1). The oblique osteotomy began from the anterior ramus border to the posterior border of the mandibular angle with surgybone devices (Silfradent S.r.l.). We achieved clean bony cuts, minimal bone loss, and heat generation. Internal distractors (Zhejiang cibei Inc) were fixed with a 2-mm diameter and 7 mm length. Raised-head self-tapping, self-drilling screws were used for neonates. A total of eight mini-screws were used, two in each footplate. After fixing distractors, the segments were easily distracted. The puncture site was performed in the postauricular area, after which a hemostat was used to pass through the puncture site to grasp the activating arm of the distractor device, and the activator was then pulled through the puncture site. The distractor was distracted, and the gap was created between two segments approximately 6-mm in size. This gap was filled with a 5 mm allogenic acellular bone (Beijing Datsing Bio-Tech co.Ltd; Figure 2A), after which the direction extension rod was reversed, making the two side mandibular decellularized bone block clamping (Figure 2B). A polyglactin 4-0 (Vicryl; Ethicon Inc) suture was used for reconstruction of the pterygomasseteric sling. Sling restoring is important for remodeling of the mandible and functional loading. The dermis was sutured approximately with a polyglactin 6-0 (Vicryl; Ethicon Inc), and skin was closed with 7-0 polypropylene (Prolene; Ethicon Inc) suture. Antibiotics intravenous drip was given for 7 days postoperatively. The jaw extending began on the 5th day. The jaw was extended 0.6 mm twice a day until the mandibular gum line was 0 to 2 mm in front of the maxillary line. After 2 weeks of the surgery, stitches were taken off. Normally, a 25-mm distractor was used. Also, either distraction to the full length of the 20 mm or overcorrection was performed to compensate for the regenerative contraction. The mandibular distractor will be removed after 3 months.

## Results

**Figure 3:**
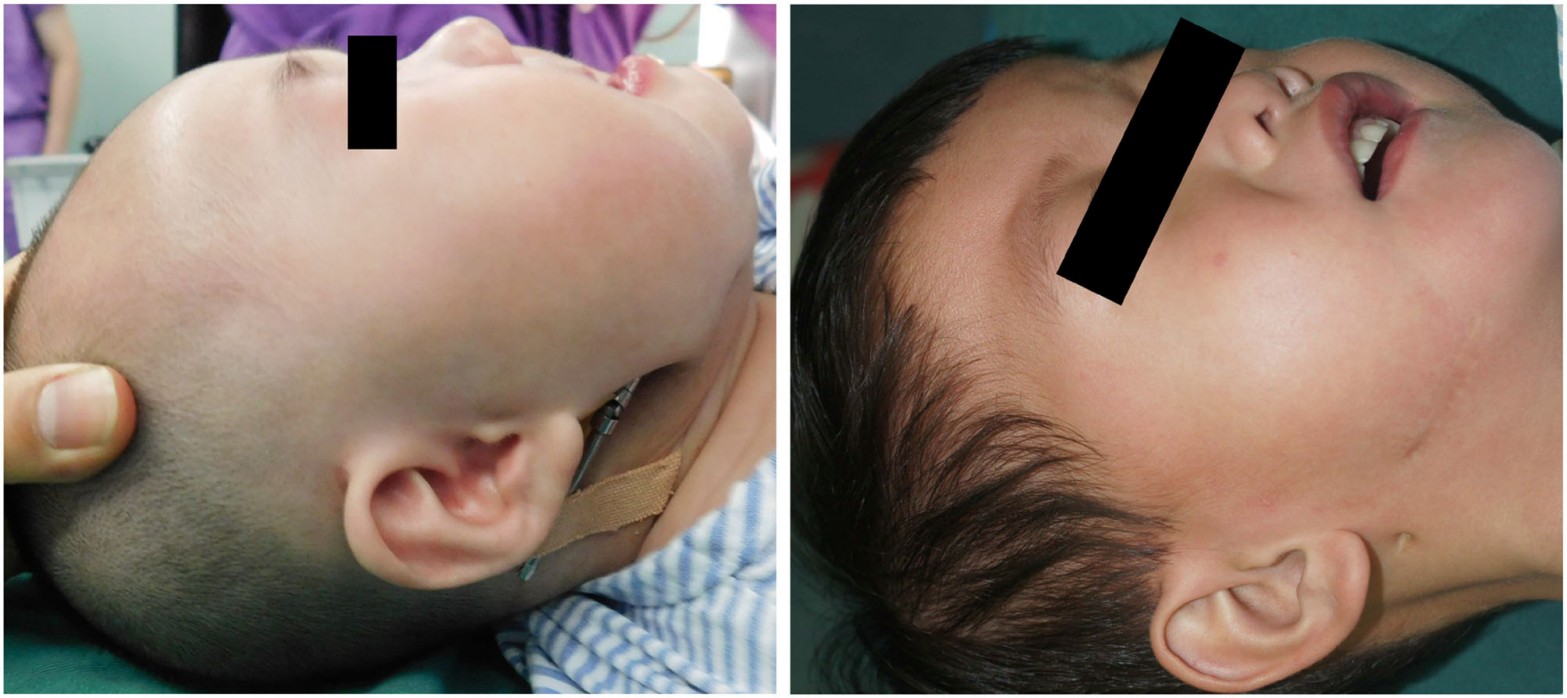
The 3 months and 1 year post-operatively.

Our patients were 428 neonates with PRS who were in need of preoperative intubation and ventilator support to maintain ventilation. The age of the patients ranged from 3 days to 28 days (mean 13.12 days, median 6.5 days). When initially evaluated, 6 neonates suffered with severe growth retardation and required nasogastric feeding until they weighed 2.5 kg. All procedures were completed in <1 h, with <20 ml blood loss. Immediately after surgery 399 patients were extubatedy, and 29 patients were extubated 4–6 days after the operation. In 48 cases, infection occurred 1 month after discharge. The total mandibular distraction length was 15–20mm. In 22 cases, soft tissue growth was found at the end of osteotomy when the distractor was removed 3 months after surgery. The mandible healed well again by excision of the interosseous soft tissue. There were 71 cases with complications that are listed in Table 2. A total of 399 patients did not need any form of supplemental oxygenation or any additional airway support after operation. 96% of patients were able to feed immediately after the procedure, while 4% of neonates could start oral feeding about 6 days after surgery. A successful distraction was a correction of the tongue from the initial vertical to a physiologically normal horizontal posture (Table 2, Figures 3A,B). The absence of endotracheal intubation in the typical preoperative photograph we provided was due to the mandibular retraction.

**Table 2:** Treatment results of all patients.

**Results**	***n***	**% Of all patients (*n* = 428)**
Intubated	428	100
Peroperative intubation	428	100
Immediate extubation after surgery	399	93.2
Extubation in 6 days postoperatively	29	6.8
Peroperation supplemental oxygen	428	100
MDO	428	100
Complication	71	16.6
Surgical-site Infections	18	4.2
Bone union partly	22	5.1
Exposed distractor	18	4.2
NI	13	3.0
Injury to the marginal mandibular branch	12	2.8
Facial nerve trunk	1	0.2
NG-tube	301	70.3
Peroperative NG-tube	301	70.3
Immediate removal after surgery	211	49.3
Removal 5 days postoperatively	90	21.0
Immediate oral feeding after surgery	410	95.8

*MDO: mandibular distraction osteogenesis; NG-tube: nasogastric tube; NI: nerve injury*.

### Case 1

**Figure 4:**
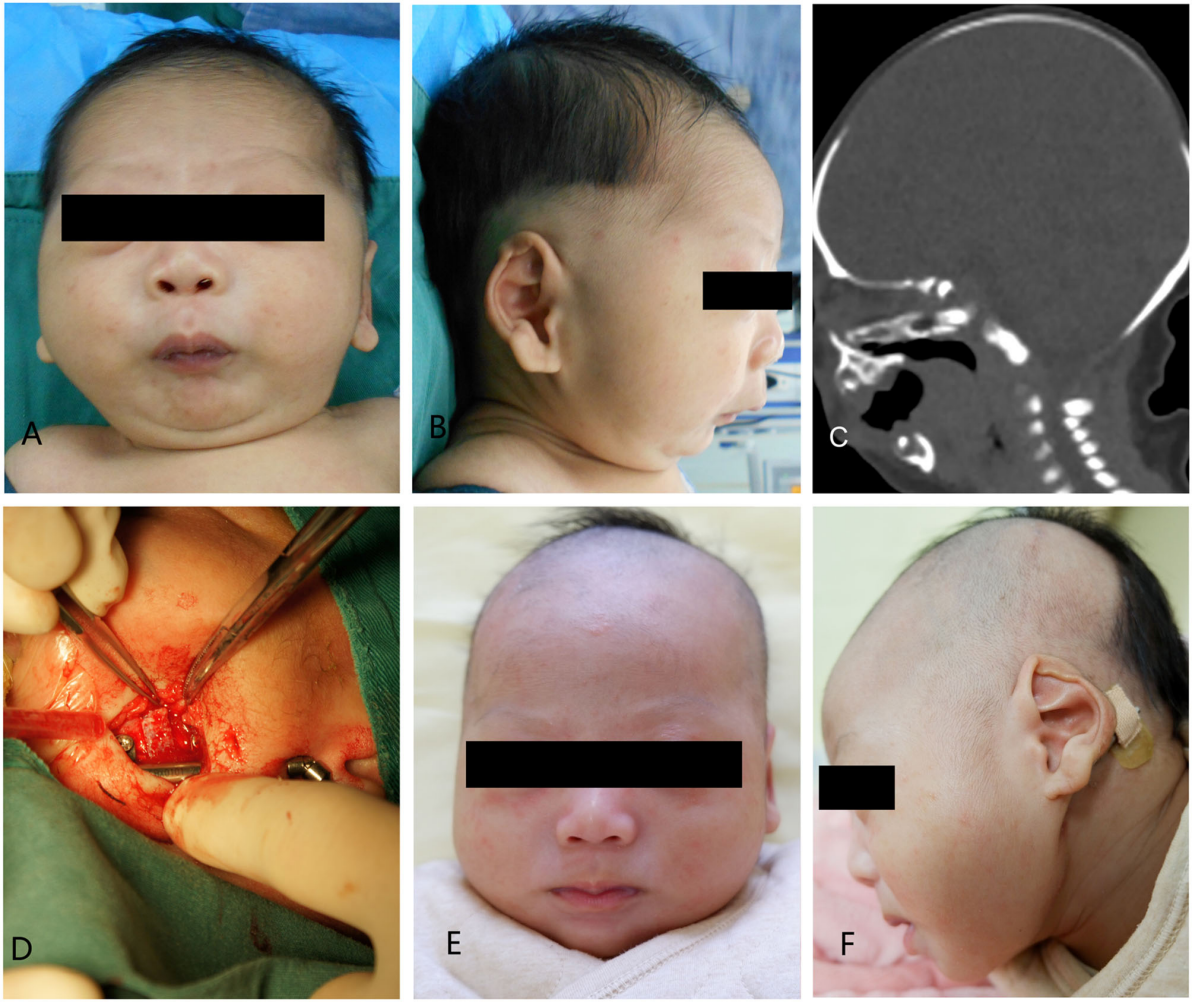
Case 1. **(A)** A preoperative frontal view. **(B)** Preoperative lateral view. **(C)** Preoperative CT-scan. **(D)** Intraoperative situation. **(E)** Postoperative anteroposterior view. **(F)** Postoperative lateral view.

A 5-day-old boy, who had a micrognathia, glossoptosis, and cleft palate, was diagnosed with PRS. 3-dimensional CT revealed severe mandibular hypoplasia leading to the compromised pharyngeal airway. The treatments were planned. Stage I included placing mandibular distractors to advance the mandible, while Stage II included removing consolidation of bilateral distractors 3 months postoperatively. In Stage I, we cut the mandible and placed the distractor based on the above-mentioned method, and extended 0.5 cm, implanting the allogenic acellular bone between the fractures during the surgery. 5–0 vicryl and 6–0 ethicon were used for extraoral closure. Both devices were activated simultaneously at a rate of 1.2 mm/day. A total distraction of 20 mm was done. Oxygen saturation increased to 95%−99% (mean 97%). The distractors were surgically removed after 3 months of consolidation. Improvement in feeding and removal of nasogastric feeding tube was performed following a mandibular distraction. Postoperative weight gain was satisfactory, and no episodes of aspiration pneumonia was observed at follow-up (Figures 4A–F).

### Case 2

**Figure 5:**
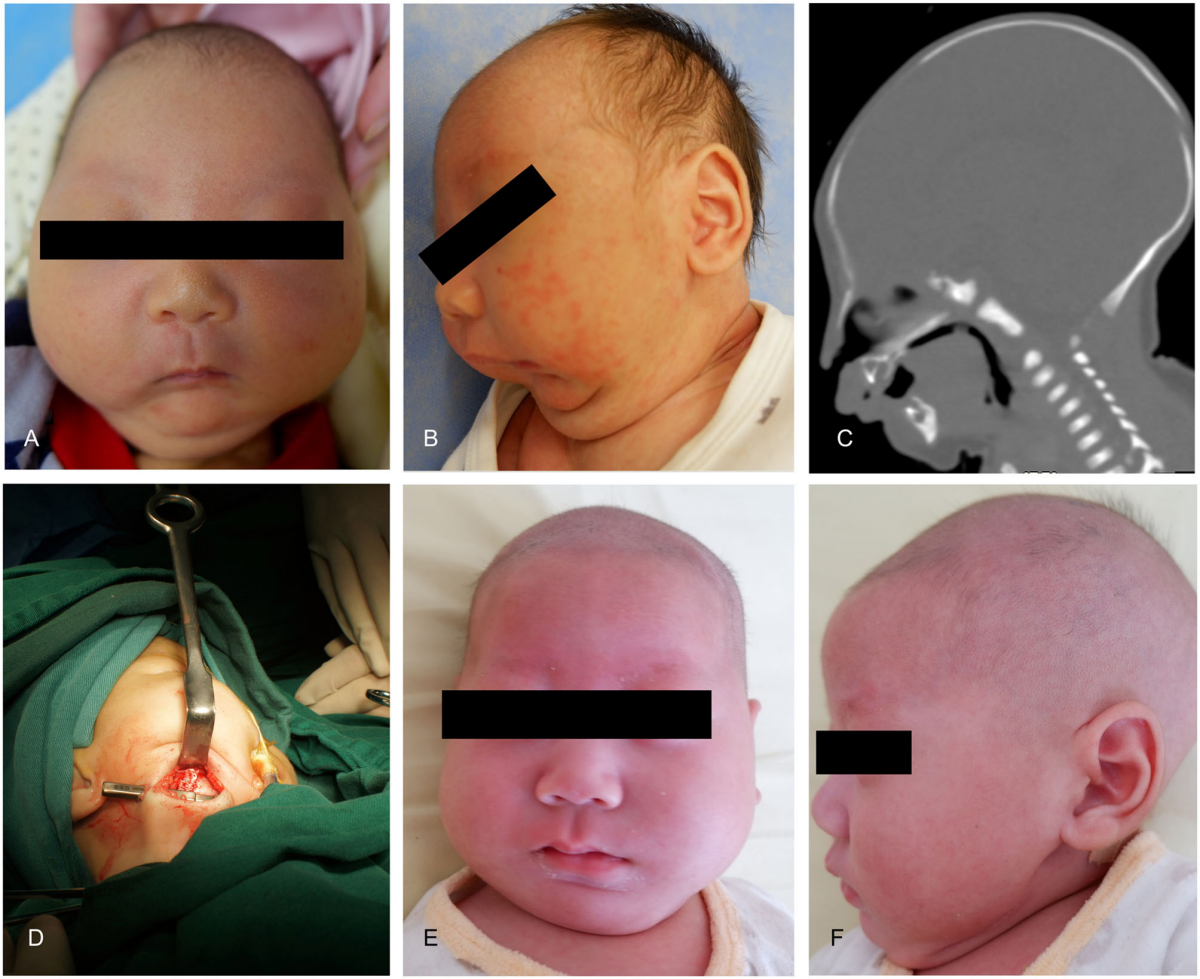
Case 2. **(A)** A preoperative frontal view. **(B)** Preoperative lateral view. **(C)** Preoperative CT-scan. **(D)** Intraoperative situation. **(E)** Postoperative anteroposterior view. **(F)** Postoperative lateral view.

The patient was a 7-day-old boy who had micrognathia, glossoptosis, and cleft palate. He had difficulties with breathing and feeding and was diagnosed with PRS. He was conservatively managed by nasogastric tube feeding and prone positioning. Severe airway obstruction was observed in this infant. Moreover, he could only receive feeding *via* the nasogastric tube. At birth, his weight was 3.1 kg. The SpO2 ranged between 80 and 85%. After admission, we decided to operate the boy. Tracheal intubation was removed after surgery. Bilateral distractors were removed under general anesthesia after 3-month of consolidation (Figures 5A–F).

## Discussion

Pierre Robin, a French stomatologist, first described the associated syndromes including micrognathia, glossoptosisglossopteris, and cleft palate in 1923. The triad was then well known as the Pierre Robin sequence (PRS) by 1974. A series of genetic mutations have been identified associating with PRS from the qualitative analysis in reported studies. However, it is still unclear if the genetic mutation is the sole cause of PRS, because varying pattern of genetic mutations were observed in numerous cases (5). The primary malformation in PRS is thought to be mandibular micrognathia, which in turn causes glossoptosis (4). There are various airway management options for patients suffering from PRS before mandibular distraction appears, such as nasopharyngeal airway placement and prone position keeping. Positive pressure mask ventilation of the airway can also be beneficial in some cases. If there is no better option, other effective alternatives include tongue-lip adhesions, glossopexy procedures, or subperiosteal release of mouth floor combined with glossopexy (6). Some of the studies suggest that the tongue-lip adhesion is effective for relieving airway obstruction that in the majority of patients with PRS is unresponsive to positioning alone (7, 8). However, this method has many complications, such as infection, dehiscence, lip scarring, and submaxillary duct obstruction. Denny et al. (9) have reported a high incidence of secondary intervention requirements with long-term follow-ups. For the most severe cases, a tracheostomy is always performed to save life. Nevertheless, the mortality rates from the tracheostomy alone, independent of the underlying diagnosis, are as high as 5% (10). As a result, Snyder et al. (11) in 1973, reported the first experimental craniofacial application of distraction osteogenesis (DO) in a canine model. McCarthy et al. (12) firstly reported gradual mandibular elongation in cases with congenital hypoplasia in 1992. Later on, prospective research of Soto et al. (13) evaluated 29 cases with tongue-based upper airway obstruction treated with mandibular DO. Decannulation or extubation was performed postoperatively in all cases. Similarly, numerous studies have verified the successfulness of DO for relieving upper airway obstruction in the management of patients with micrognathia (14, 15). Zhang et al. (16) think cases with PRS and preoperative smaller gonial angle or postoperative pulmonary infection may be more likely to undergo prolonged mechanical ventilation after MDO. For others, extubation may be attempted within 6 days after MDO. Jiayu et al. (17) think bilateral mandible distraction could improve the nutrition status of PRS infants. These studies suggest that DO is an effective approach for achieving mandibular advancement. Mandibular DO avoids a tracheostomy. Mandibular distraction has been an effective way to treat PRS patients, avoiding tracheostomy, in 90%−95% of cases (18). Mandibular DO can increase the cavity of pharynges, relieving breathing, and feeding problems. As the cavity slowly increases, it is impossible to immediately remove endotracheal tube or tracheotomy, which is a limitation of DO in treating neonatal micrognathia. This procedure provides only a gradual improvement in upper airway. Consequently, ordinarily DO cases could not be extubated immediately after an operation and could start feeding 5–8 days post-operatively. Children who are extubated for a long time may be at high risk of airway straitness and respiratory tract infection. Due to hospital expenses but also personal safety, DO techniques and approaches should be further improved.

In this paper, we proposed a new method, which consists of placing allogenic acellular bone into the gap extending some 6 mm in size, equal to distraction for 5–8 days. In this way, we solve the breathing problem right post-operation, and feeding problem during following distraction days. Nonetheless, placing allogenic acellular bone matrix and mandibular distraction osteogenesis can lead to some complications, such as nonunion of the mandible, infection of the allogenic acellular bone, inferior alveolar nerves damage, dislodgement of pins or distractors, distraction failure, and tooth bud damage. Consequently, it is important to consider the correct indications, careful operative procedure, and appropriate size of the allogenic acellular bone (usually 0.5 mm) to avoid adverse events. The proposed procedure is safe and allows for successful extubating immediately post-operation.

In conclusion, bilateral mandibular distraction osteogenesis and placing allogenic acellular bone in the neonate are safe and accurate procedures, which are the primary treatment options for cases of severe PRS. It can be considered that the tension of distraction can promote osteogenesis in acellular bone and thus improve distractive effect of the mandible. The advantage of our technique is immediately performing extubating right after the operation and instantly relieving the difficulty in breathing. The feeding problem may also be solved earlier than with conventional distraction.

## Data Availability Statement

The raw data supporting the conclusions of this article will be made available by the authors, without undue reservation.

## Ethics Statement

The studies involving human participants were reviewed and approved by Ethics Committee of the Children's Hospital of Nanjing Medical University. Written informed consent to participate in this study was provided by the participants' legal guardian/next of kin. Written informed consent was obtained from the minor(s)' legal guardian/next of kin for the publication of any potentially identifiable images or data included in this article.

## Author Contributions

WS revised the manuscript and approved the final manuscript as submitted. JC, YJ, and LK performed the surgery and conducted the data analyses. LS and JC performed postoperative follow-up and analyzed the data. TH wrote a draft of the article and edited the figures.

## Funding

This study was supported by Nanjing Medical Science and Technology Development Foundation (grant no. ZKX21045).

## Conflict of Interest

The authors declare that the research was conducted in the absence of any commercial or financial relationships that could be construed as a potential conflict of interest.

## Publisher's Note

All claims expressed in this article are solely those of the authors and do not necessarily represent those of their affiliated organizations, or those of the publisher, the editors and the reviewers. Any product that may be evaluated in this article, or claim that may be made by its manufacturer, is not guaranteed or endorsed by the publisher.
